# Long-Term Follow-Up Results of Fecal Microbiota Transplantation for Irritable Bowel Syndrome: A Single-Center, Retrospective Study

**DOI:** 10.3389/fmed.2021.710452

**Published:** 2021-07-30

**Authors:** Jiaqu Cui, Zhiliang Lin, Hongliang Tian, Bo Yang, Di Zhao, Chen Ye, Ning Li, Huanlong Qin, Qiyi Chen

**Affiliations:** Intestinal Microenvironment Treatment Center of General Surgery, Shanghai Tenth People's Hospital, Tenth People's Hospital of Tongji University, Shanghai, China

**Keywords:** irritable bowel syndrome, fecal microbiota transplantation, efficacy, safety, retrospective study

## Abstract

**Objective:** This study aimed to investigate the long-term efficacy of fecal microbiota transplantation (FMT) in patients with irritable bowel syndrome (IBS).

**Study Methods:** In this single-center long-term follow-up study, FMT treatment was administered to patients with moderate to severe IBS (IBS severity scoring system (IBS-SSS) > 175). After 1 year of treatment, it was decided whether to repeat FMT based on IBS-SSS score (IBS-SSS > 175). Baseline characteristics before and after FMT and questionnaires were completed at 1, 3, 6, 12, 24, 36, 48, and 60 months after FMT. The study outcomes included treatment efficacy rates, change of IBS-SSS, IBS-specific quality of life and fatigue, effect on stool frequency, Bristol Stool Scale for IBS-C and IBS-D, and side effects.

**Results:** A total of 227 patients (47.58% IBS-C, 39.21% IBS-D, and 13.22% IBS-M) were recruited (142 females and 85 males with a mean age of 41.89 ± 13.57 years). The efficacy rates were 108 (51.92%), 147 (74.62%), 125 (74.41 %), 88 (71.54%), 78 (75.00%), 65 (73.03%), 45 (61.64%), and 37 (62.71%) at different follow-up time points. The total IBS-SSS score was 321.37 ± 73.89 before FMT, which significantly decreased after 1 month. The IBS-specific quality of life (IBS-QoL) score was 40.24 ± 11.34 before FMT, increased gradually, and was significantly higher at 3 months compared to before FMT. The total Fatigue Assessment Scale (FAS) score was 47 ± 8.64 before FMT and was significantly lower at 3 months. During follow-up, 89 (39.21%) side effects occurred that were alleviated by symptomatic treatment, and no serious adverse events were detected.

**Conclusion:** Based on 60 months of long-term follow-up, the safety and efficacy of FMT for IBS was established. However, as the treatment effect declines over time, periodic and repetitive FMT is required for a sustained effect.

## Introduction

Irritable bowel syndrome (IBS) is one of the most commonly diagnosed gastrointestinal (GI) conditions. It is a symptom-based condition defined by the presence of abdominal pain or discomfort, with altered bowel habits, in the absence of any other disease to cause these sorts of symptoms (1). The prevalence of IBS in the global population ranges from 5.7 to 34% (2, 3), and in Southeast Asia, it is relatively infrequent (7.0%) (2). With the rapid economic growth and current environmental changes, the incidence of IBS in China has been on the rise year by year, showing a 5–10% prevalence in adults (4).

While medical treatment for IBS is still limited, the overall illness burden is high, patients report a low quality of life, low work efficiency and absenteeism in the workplace, and significant direct and indirect healthcare costs (5). The etiology of IBS is not fully understood, and there is no effective treatment for the condition. Current evidence suggests that the microbiota of the GI tract could be a significant factor in the etiology of IBS (6). The gut microbiota of patients with IBS differs from that of healthy subjects, with the former having a lower bacterial diversity (dysbiosis), for example (7, 8). It is speculated that changes in the intestinal environment will lead to an imbalance in the composition of gut microbiota, termed “dysbiosis,” which has been associated with the occurrence of IBS (9). Consequently, probiotics and antibiotics have been studied as a potential treatment option for IBS (10, 11); however, the reported magnitude of improvement in associated symptoms was limited.

Fecal microbiota transplantation (FMT), also known as fecal bacteriotherapy or fecal infusion, consists of administration of a liquid filtrate of feces from a healthy donor into the GI tract of a recipient individual (12). In recurrent *Clostridioides difficile* infections, FMT has shown excellent effects. The cure rate of FMT is higher than conventional treatment with antibiotics (13, 14), and studies have shown that FMT can restore intestinal microbial balance in treated patients (13, 15). Using the FMT method, our team has treated 2,010 cases of various GI dysfunction diseases, including IBS. The long-term (36 months) effective rate has exceeded 60% (16). A number of short-term follow-up studies with small sample sizes showed that FMT can improve symptoms and restore the intestinal microbiota diversity in IBS patients (17–19). The current study retrospectively analyzed the long-term efficacy of FMT in IBS by applying a large sample size and conducting a 5-year follow-up period. Furthermore, the differences in efficacy between various transplantation approaches were compared.

## Materials and Methods

### Participants and Study Design

In this single-center, retrospective study, consecutive patients treated at the Intestinal Microenvironment Diagnosis and Treatment Center, Tenth People's Hospital of Tongji University (Shanghai, China), between January 2014 and January 2019 were included if they met the following criteria: (1) aged 18–65 years and complied with the diagnostic criteria of Rome III or Rome IV; (2) had moderate to severe disease activity (IBS severity scoring system (IBS-SSS) ≥ 175); (3) had normal colonoscopy (performed within 1 year) if the patient was ≥40 years or had blood in the stool; and (4) had no response shown to conventional treatment for IBS. The exclusion criteria were as follows: (1) other chronic GI diseases; (2) fecal sample positive for enteropathogenic microorganisms; (3) positive screening for HIV, HBV, or HCV antibodies; (4) a history of surgical interventions in the GI region (except for appendectomy, hernia repair, cholecystectomy, and gynecological or urological procedures); (5) severe psychiatric disorders; (6) fecal calprotectin ≥50mg/kg; (7) severe allergies or asthma; (8) abnormal biochemistry screening result; (9) abnormal colonoscopy findings; (10) pregnancy, planned pregnancy, or breastfeeding females; (11) ingestion of probiotics or antibiotics <4 weeks prior to inclusion; (12) immunocompromised patients or those using immunosuppressive drugs; and (13) GI or systemic malignancies.

The data used in this study were obtained from the follow-up system of the Intestinal Microenvironment Diagnosis and Treatment Center, Tenth People's Hospital of Tongji University, Shanghai, China. All patients were checked during study visits for baseline (before FMT) and 1, 3, 6, 12, 24, 36, 48, and 60 months. At the end of the follow-up period, they completed the IBS-SSS and IBS-specific quality of life (IBS-QoL) questionnaire. Additional questionnaires included the following: Bristol Stool Form Scale, stool frequency, and Fatigue Assessment Scale (FAS). Any complications within 7 days after the first transplantation were recorded. Adverse events were evaluated by the use of the modified Common Terminology Criteria for Adverse Events version 3.0 (20). All enrolled patients signed the FMT treatment informed consent.

### The Donor Screening

A total of 19 fecal donors were recruited for this study. Once enrolled, full-time donor managers were employed to manage the diet, lifestyle, and physical condition of the donors during the collecting period. All donors were screened according to guidelines (21, 22) and were recruited based on the following inclusion criteria: (1) 18–30 years of age; (2) good previous and current health status; (3) normal body weight (body mass index (BMI) between 18 and 22 kg/m^2^); (4) normal bowel movements (defined as one to two times per day and type 3–4 on the Bristol Stool Form Scale); and (5) no medications taken. The exclusion criteria were as follows: (1) history of antibiotic treatment within 3 months preceding donation; (2) history of intrinsic GI illnesses; and (3) metabolic syndromes, obesity, or any ongoing diseases. A single universal donor was recruited for our trial, who was a 24-year-old healthy University student. For the purposes of informed consent, the donor was required to be over 18 years of age. Current guidelines recommend using a donor questionnaire that is similar to current protocols for screening blood donors. Blood collection was performed before FMT donation, which included a complete blood count, chemistry, and iron profile. The donor blood sample was negative for common viruses (hepatitis A, B, and C; HIV-1 and HIV-2; cytomegalovirus; Epstein–Barr virus; herpes simplex; and varicella zoster) and *Treponema pallidum*. The donor feces were negative for common enteric pathogens (*Yersinia* spp., *Salmonella* spp., *Shigella* spp., *Campylobacter jejuni, Clostridioides difficile* toxin, helminths, ova, parasites, and *Helicobacter pylori*). Multidrug-resistant bacteria were determined using standard screening methods.

### Preparation of FMT

#### Preparation of Fresh FMT Solution

According to the fresh FMT solution preparation method previously established by our team (23), fresh stool (200 g) was immediately mixed in a blender with 500 ml 0.9% sterile saline for several seconds until it developed a smooth consistency. The obtained stool suspension was filtered several times through gauze screens with decreasing apertures (2.0–0.7 ± 0.2 mm) to remove large and small particles that could clog the nasointestinal tube. The resulting concentrated fecal bacterial suspension was either administered to the patient without delay or amended with glycerol to a final concentration of 10%. The latter suspension was stored frozen at −20°C for 1–4 weeks until further use. The stool suspension was poured into a sterile bottle for administration within 2 h. The study used standardized, processed stool from the same universal donor and the same amount of stool for FMT for each patient.

#### Preparation of Freeze-Dried FMT Capsules

The FMT capsules were prepared according to the method previously established by our team (24). After the preparation of the above fresh FMT solution, centrifugation was carried out at 4°C, the supernatant was removed, and freeze-drying protectant was added. The bacterial suspension was mixed well with an oscillator, prepared for pre-freezing, and the frozen sample was quickly transferred to the freeze dryer for freeze-drying. Finally, the freeze-dried powder was put into acid-resistant hydroxypropyl methylcellulose. The capsules were sealed and stored at −20°C (48 capsules/200 g feces).

#### FMT Procedure

An initial dose of oral antibiotic (500 mg vancomycin orally twice per day) was administered for 3 consecutive days. The day before FMT, polyethylene glycol was administered orally or through a nasointestinal tube to prepare the bowel. Patients received fresh FMT for 6 consecutive days through a nasointestinal tube or colonoscopy. Altogether, 100 g of stool suspension was administered through the nasointestinal tube or colonoscopy within 6 min daily for 6 consecutive days. Meanwhile, patients who could not tolerate the nasointestinal tube or endoscopic approach received four capsules twice daily on an empty stomach for 6 consecutive days. The nasointestinal tube was flushed with 50 ml of saline solution before and after each procedure to ensure that the entire volume of stool suspensions was transplanted into the intestine. For the capsule group, the 48 capsules contained sieved, concentrated, and freeze-dried powders derived from 200 g of donor stool.

Twelve months after FMT treatment, the total IBS-SSS score of the annual follow-up results was used to decide whether the FMT treatment would be continued. If this score decreased by more than 50 but was still over 175 after FMT, it was suggested that FMT should be continued. On the contrary, if the total IBS-SSS score after FMT was <175, no further treatment was considered necessary. If the total IBS-SSS score after FMT had no obvious change or increase, the FMT treatment was set to be stopped, and conventional treatment would be adopted.

### Questionnaires

This study used the questionnaires discussed below. All steps were completed under the direct supervision of the investigators to ensure that participants understood and completed all questions. All questionnaires were formally translated to Mandarin Chinese and validated. Abdominal symptoms were assessed using the IBS-SSS questionnaires, which included five dimensions: abdominal distension/bloating, abdominal pain frequency, abdominal pain severity, satisfaction with bowel habits, and quality of life. Fatigue was evaluated on the FAS. Quality of life was determined using the IBS-QoL questionnaires, where higher IBS-QoL scores indicated a better quality of life. Patients whose total IBS-SSS score decreased by ≥50 points after FMT were considered responders. A decrease of ≥175 points in the IBS-SSS total score, a decrease of ≥4 points in the FAS score, and an increase of ≥14 points in the IBS-QoL score were considered to indicate significant clinical improvements in abdominal symptoms, fatigue, and quality of life, respectively (25). The fulfillment of all these criteria at the same time was considered effective in the treatment of IBS by FMT.

### Statistical Methods

Statistical analysis was performed by descriptive methods and SPSS 20.0 software. The count data were expressed by the number of cases (%), and the measurement data that conform to the normal distribution were expressed by *x* ±*s*. A chi-square test or Fisher's exact probability method was used to compare the treatment efficacy rate between groups. The comparison of time points before and after treatment was performed by univariate analysis of variance. The IBS-QoL score was transformed into a 0–100 scale using the following formula: total score = (sum of the items – 34/170) × 100.

## Results

### Patient Characteristics

**Figure 1:**
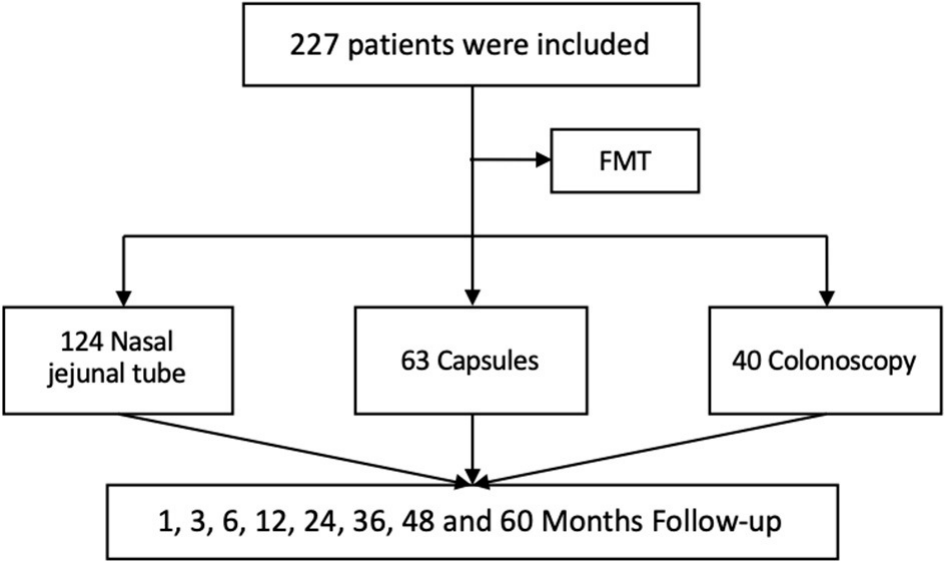
Flowchart of patient inclusion. FMT, fecal microbiota transplantation.

A total of 227 patients were enrolled in this study (Figure 1), including 142 females and 85 males with a median age of 41.89 ± 13.57 years, BMI of 20.86 ± 1.63, and weight of 61.33 ± 10.58 kg. According to the classification of IBS, there were 108 (47.58%) constipation-predominant IBS (IBS-C), 89 (39.21%) diarrhea-predominant IBS (IBS-D), and 30 (13.22%) mixed-type IBS (IBS-M) cases. The history of IBS-related drug use included laxatives (132, 58.15%), prokinetic drugs (84, 37%), antidiarrheal drugs (106, 47%), psychotropic drugs (97, 42.73%), painkillers (65, 28.63%), PPI (183, 80.62%), antibiotics (152, 66.96%), probiotics (197, 86.78%), traditional Chinese medicine (118, 51.98%), and spasmolytic agents (149, 65.64%). The total scores of IBS-SSS, IBS-QoL, and FAS were 321.37 ± 73.89, 40.24 ± 11.34, and 47 ± 8.64, respectively, before FMT. According to the transplantation method, 124 (54.63%) patients received the transplant through a nasointestinal tube, 63 (27.75%) in the form of oral capsules, and 40 (17.62%) through colonoscopy. The average course of FMT was 3.93 ± 2.30, including 3.83 ± 1.78 for nasointestinal tube, 5.19 ± 2.94 for capsule, and 2.25 ± 1.24 for colonoscopy (Table 1).

**Table 1:** Baseline characteristics of patients.

**Characteristics**	**Overall**
*n*	227
Age (mean ± SD)	41.89 ± 13.57
Sex, female/male	142/85
BMI (mean ± SD)	20.86 ± 1.63
Weight (mean ± SD)	61.33 ± 10.58
Type of IBS (%)	
IBS with constipation	108 (47.58)
IBS with diarrhea	89 (39.21)
IBS mixed	30 (13.22)
History of IBS-related medications (%)	
Laxatives	132 (58.15)
Prokinetic drugs	84 (37)
Antidiarrheal	106 (47)
Psychotropic drugs	97 (42.73)
Painkillers	65 (28.63)
PPI	183 (80.62)
Antibiotics	152 (66.96)
Probiotics	197 (86.78)
Traditional Chinese medicine	118 (51.98)
Spasmolytic	149 (65.64)
IBS-SSS score (mean ± SD)	321.37 ± 73.89
IBS-QoL score (mean ± SD)	40.24 ± 11.34
FAS score (mean ± SD)	47 ± 8.64
FMT pathway (%)	
Capsules	63 (27.75)
Nasointestinal tube	124 (54.63)
Colonoscopy	40 (17.62)
Average course of FMT (times)	3.93 ± 2.30
Nasointestinal tube (times)	3.83 ± 1.78
Capsules (times)	5.19 ± 2.94
Colonoscopy (times)	2.25 ± 1.24

*BMI, body mass index; FMT, fecal microbiota transplantation; IBS-QoL, IBS-specific quality of life; IBS-SSS, IBS severity scoring system; PPI, proton pump inhibitor*.

### Rate of Effective Follow-Up

In this study, a total of 227 patients were enrolled. Based on 60 months of long-term follow-up data, the effective follow-up rates at 1, 3, 6, 12, 24, 36, 48, and 60 months after FMT were 51.92% (108/208), 74.62% (147/197), 74.41% (125/168), 71.54% (88/123), 75.00% (78/104), 73.03% (65/89), 61.64% (45/73), and 62.71% (37/59), respectively.

### Effect of Different Transplantation Routes on the Treatment Efficacy

Three transplantation groups were included in this study: the nasointestinal tube group (*n* = 124), capsule group (*n* = 63), and colonoscopy group (*n* = 40).

The effective follow-up rates at 1, 3, 12, and 60 months, respectively, were 60 (53.10%), 80 (74.07%), 48 (70.59%), and 23 (60.53%) for the nasointestinal tube group; 31 (54.39%), 43 (78.18%), 30 (83.33%), and 12 (75.00%) for the capsule group; and 17 (44.74%), 24 (70.59%), 10 (52.63%), and 2 (40.00%) for the colonoscopy group (Table 2). A significant difference in the efficacy rates among the three groups was observed only at 36 months after FMT.

**Table 2:** The effect of different transplantation routes on efficacy.

**Follow-up time**	**Nasointestinal tube group (** ***n*** **=** **124)**		**Capsules group (** ***n*** **=** **63)**		**Colonoscopy group (** ***n*** **=** **40)**		**χ^**2**^**	***p***
	**No**.	**Effective number (%)**	**No**.	**Effective number (%)**	**No**.	**Effective number (%)**		
1 month	113	60 (53.10)	57	31 (54.39)	38	17 (44.74)	0.987	0.61
3 months	108	80 (74.07)	55	43 (78.18)	34	24 (70.59)	0.677	0.713
6 months	92	68 (73.91)	47	39 (82.98)	29	18 (62.07)	4.143	0.126
12 months	68	48 (70.59)	36	30 (83.33)	19	10 (52.63)	5.826	0.054
24 months	61	44 (72.13)	31	27 (87.10)	12	7 (58.33)	4.465	0.107
36 months	53	37 (69.81)	26	23 (88.46)	10	5 (50.00)	6.116	0.047^*^
48 months	47	28 (59.57)	20	14 (70.00)	6	3 (50.00)	1.02	0.601
60 months	38	23 (60.53)	16	12 (75.00)	5	2 (40.00)	2.214	0.331

* *p < 0.05*.

### Long-Term Follow-Up of IBS-SSS

**Figure 2:**
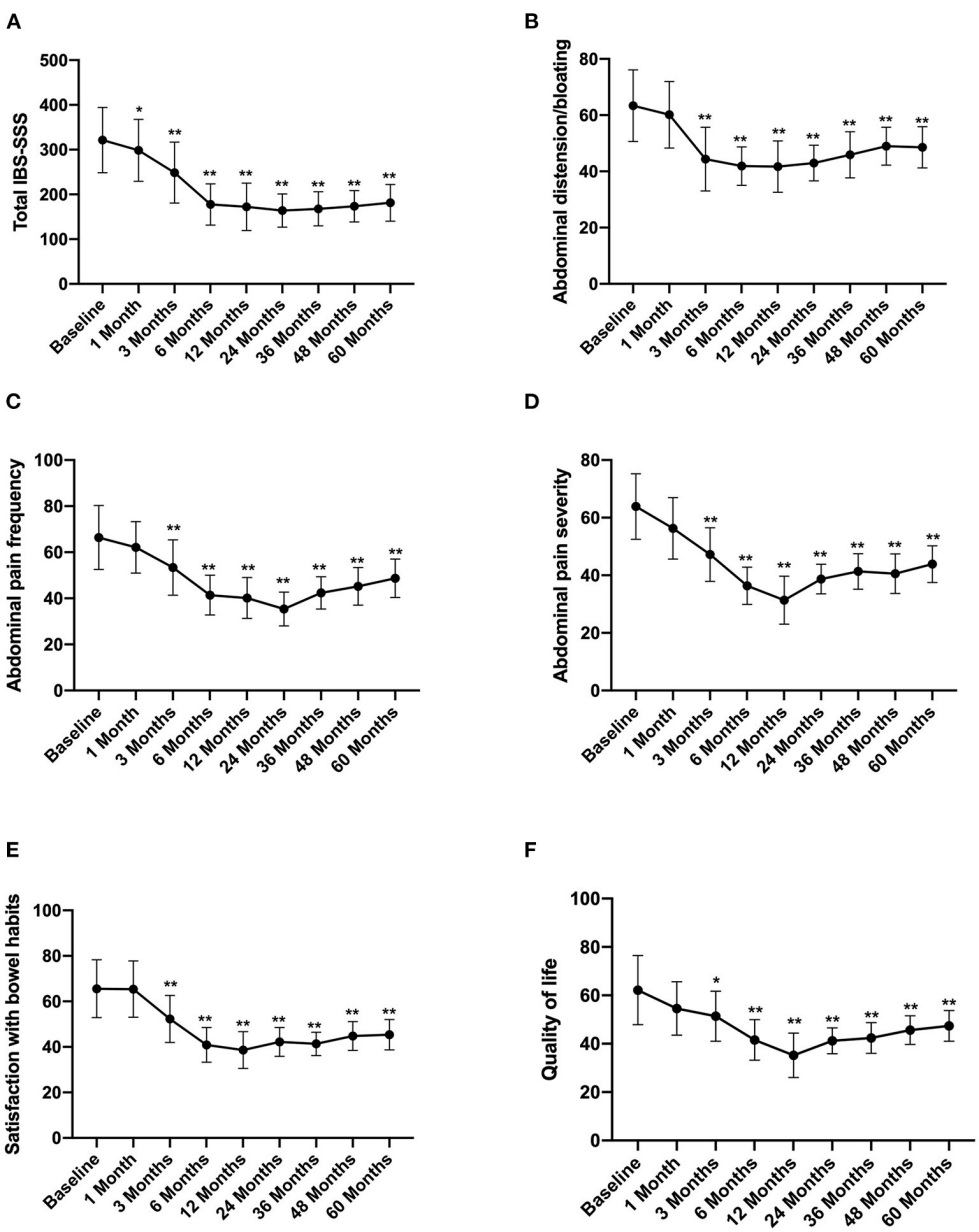
IBS-SSS score between groups and the change over time. Difference was compared between each time point of follow-up and before FMT (baseline). **(A)** Total IBS-SSS score; **(B)** abdominal distension/bloating score; **(C)** abdominal pain frequency score; **(D)** abdominal pain severity score; **(E)** satisfaction with bowel habits score; **(F)** quality of life score. IBS-SSS, IBS severity scoring system. Data are presented as *x* ± *s*, statistical analyses: univariate analysis of variance, *p < 0.05, **p < 0.01.

According to the long-term follow-up research data, after FMT, the abdominal symptoms assessed by the IBS-SSS questionnaires were significantly reduced. The total IBS-SSS score was 321.37 ± 73.89 before FMT, which significantly decreased after 1 month of FMT to 298.57 ± 69. Moreover, abdominal distension bloating, abdominal pain, and abdominal pain severity also decreased, whereas satisfaction with bowel habits and quality of life improved after 1 month of FMT (Figure 2).

### Long-Term Follow-Up of IBS-QoL

**Figure 3:**
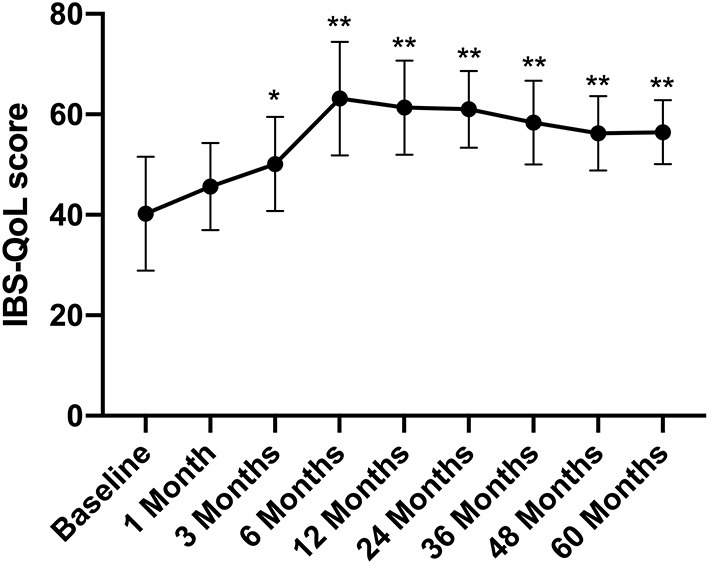
IBS-QoL score between groups and the change over time. Difference was compared between each time point of follow-up and before FMT (baseline). IBS-QoL, IBS-specific quality of life. Data are presented as *x* ± s, statistical analyses: univariate analysis of variance, *p < 0.05, **p < 0.01.

The IBS-QoL score gradually increased after FMT, rising from 40.24 ± 11.34 before FMT to 50.13 ± 9.34 at 3 months after treatment (*p* < 0.05) (Figure 3).

### Long-Term Follow-Up of FAS

**Figure 4:**
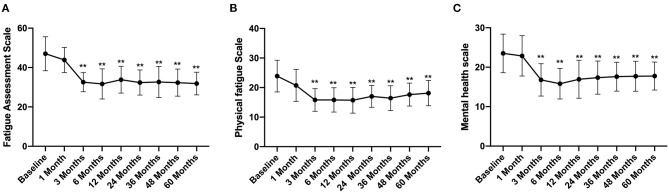
FAS score between groups and their change over time. Difference was compared between each time point of follow-up and before FMT (baseline). **(A)** total score; **(B)** physical fatigue; **(C)** mental health. FAS, Fatigue Assessment Scale. Data are presented as *x* ± *s*, statistical analyses: univariate analysis of variance, **p < 0.01.

The total FAS score was 47 ± 8.64 before FMT, which decreased gradually after FMT and was significantly lower at 3 months after FMT (32.58 ± 4.86) than that before FMT (Figure 4A). At the same time, the physical fatigue and mental health scale scores also reduced significantly at 3 months after FMT, with scores of 15.89 ± 3.86 and 16.78 ± 4.1, respectively (Figures 4B,C) and then remained at a stable level. At the 5th year of follow-up, the total FAS, physical fatigue scale, and mental health scale scores were significantly lower than those before FMT, with values of 31.89 ± 5.74, 18.12 ± 4.28, 17.77 ± 3.55, respectively (*p* < 0.01) (Figure 4).

### Change of Stool Frequency and Bristol Stool Scale for IBS-C and IBS-D

**Figure 5:**
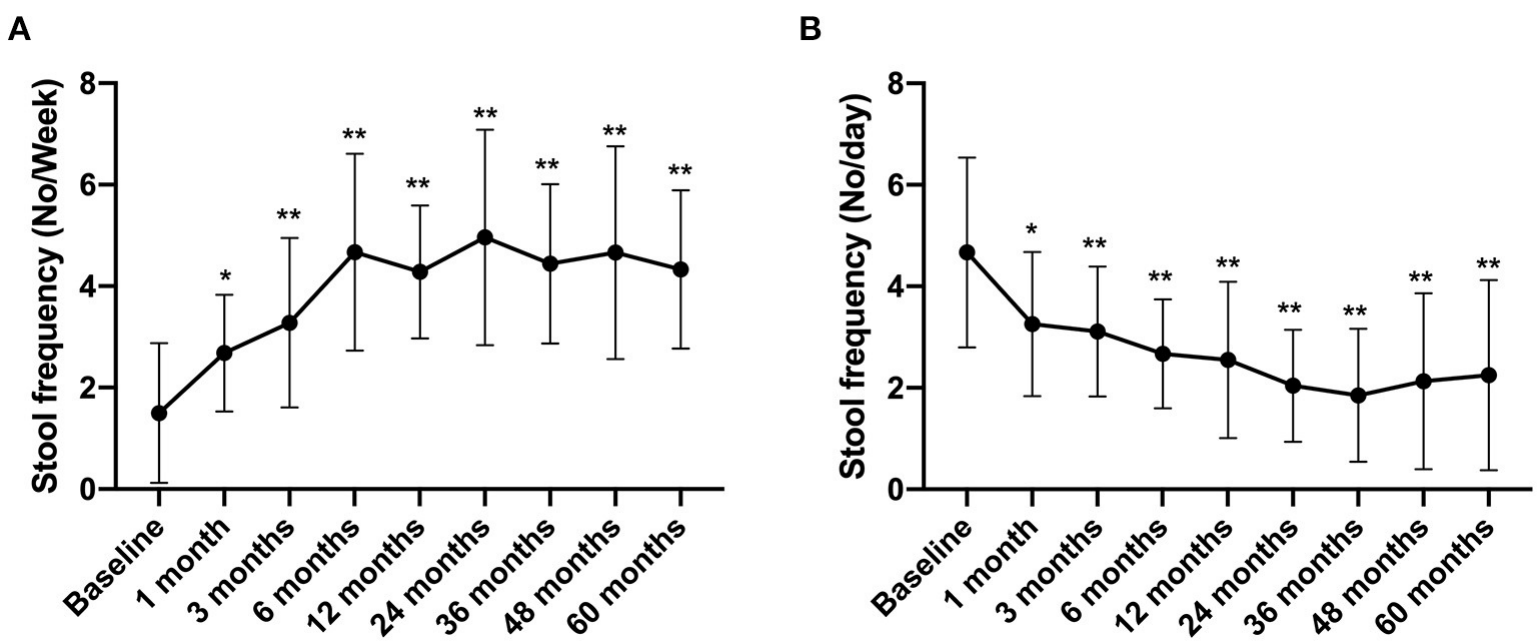
Stool frequency between groups and their change over time. Difference was compared between each time point of follow-up and before FMT (baseline). **(A)** IBS-C; **(B)** IBS-D. Data are presented as *x* ± *s*, statistical analyses: univariate analysis of variance, *p < 0.05, **p < 0.01.

In this study, patients with IBS-C and IBS-D were followed up to evaluate the stool frequency and the Bristol Stool Scale score. The results showed that the stool frequency of IBS-C patients increased from 1.5 ± 1.38 times per week before treatment to 2.68 ± 1.15 times per week at 1 month after FMT treatment (compared with that before FMT, *p* < 0.05) and increased to 4.33 ± 1.56 times per week in the 5th year after FMT (compared with that before FMT, *p* < 0.01) (Figure 5A). In contrast, the stool frequency of IBS-D patients decreased from 4.67 ± 1.87 times per day before treatment to 3.26 ± 1.42 times per day at 1 month after FMT treatment. By the 5th year, this reduced to 2.25 ± 1.87 times per day (compared with that before FMT, *p* < 0.01) (Figure 5B).

**Figure 6:**
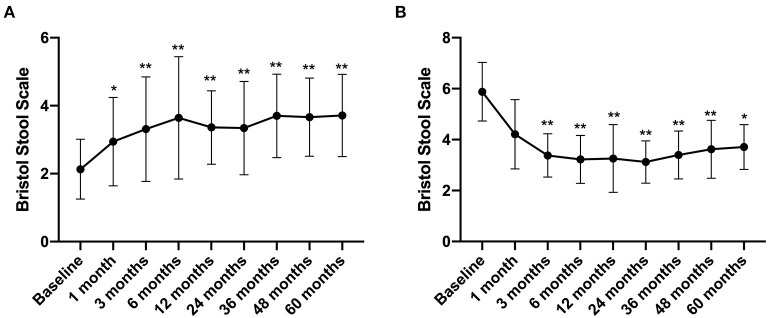
Bristol Stool Scale between groups and their change over time. Difference was compared between each time point of follow-up and before FMT (baseline). **(A)** IBS-C; **(B)** IBS-D. Data are presented as *x* ± s, statistical analyses: univariate analysis of variance, *p < 0.05, **p < 0.01.

The Bristol Stool Scale score of IBS-C patients increased from 2.13 ± 0.88 before treatment to 2.94 ± 1.3 at 1 month after FMT treatment (compared with that before FMT, *p* < 0.05) and further increased to 3.71 ± 1.21 by the 5th year after FMT (compared with that before FMT, *p* < 0.01) (Figure 6A). In contrast, the Bristol Stool Scale score of IBS-D patients reduced from 5.88 ± 1.15 before FMT to 3.38 ± 0.85 at 3 months after FMT treatment. By the 5th year, this declined to 3.71 ± 0.88 (compared with that before FMT, *p* < 0.01) (Figure 6B).

### Side Effects of FMT

Any side effects directly related to and during FMT treatment and within 1 week after FMT were considered to be adverse effects of FMT. At the same time, different side effects were observed for different FMT pathways (colonoscopy, nasointestinal, and capsule). A total of 89 (39.21%) adverse reactions occurred during follow-up. Of these, 83 were mild, and no interventions or medications were indicated (grade 1). The other six adverse events were classified as grade 2 effects. No serious adverse reactions (grade 3 or above) were observed. The main adverse events were abdominal pain in 15 (6.61%) patients, of which six (15%) with the highest incidence were in the colonoscopy group; thus, this event may be related to the colonoscopy procedure. Furthermore, seven (5.65%) and two (3.18%) cases were in the nasointestinal tube and capsule group, respectively. Of the 17 cases of abdominal distension/bloating, eight (6.45%) occurred in the nasointestinal tube pathway, three (4.76%) in the capsule pathway, and six (15%) in the colonoscopy pathway. Diarrhea presented in 13 cases, including five (12.50%) in the colonoscopy group, six (4.84%) in the nasointestinal tube group, and two (3.18%) in the capsule group. The highest incidence rate of diarrhea in the colonoscopy pathway may be related to the stimulation effect of colonoscopy. There were 16 cases of nausea, of which 11 (8.87%) occurred in the nasointestinal tube pathway, 1 (1.59%) in the capsule pathway, and 4 (10%) in the colonoscopy pathway. Of the 10 cases of vomiting, six (4.84%) occurred in the nasointestinal tube group, two (3.18%) in the capsule group, and two (5%) in the colonoscopy group. Headache occurred in seven (3.08%) cases, a single case of GI bleeding occurred after colonoscopy, and allergic reactions were detected in two (0.88%) cases, namely, one (0.81%) in the nasointestinal tube group and one (2.5%) in the colonoscopy group. Fever occurred in eight (3.52%) cases, of which five (4.03%) were in the nasointestinal tube group, two (5%) were in the colonoscopy group, and one (1.59%) was in the capsule group. No significant differences were observed concerning the adverse events among the three groups. All symptoms were cured by symptomatic treatment, and no serious adverse events were reported during treatment or follow-up (Table 3).

**Table 3:** Side effects of FMT.

	**Nasointestinal tube (124)**	**Capsules (63)**	**Colonoscopy (40)**	**Total complications (%)**	***p***
Abdominal pain (%)	7 (5.65)	2 (3.18)	6 (15)	15 (6.61)	0.06
Abdominal distension/bloating (%)	8 (6.45)	3 (4.76)	6 (15)	17 (7.49)	0.133
Diarrhea (%)	6 (4.84)	2 (3.18)	5 (12.50)	13 (5.73)	0.125
Nausea (%)	11 (8.87)	1 (1.59)	4 (10)	16 (7.05)	0.094
Vomiting (%)	6 (4.84)	2 (3.18)	2 (5)	10 (4.41)	0.828
GI bleeding (%)	0 (0)	0 (0)	1 (2.5)	1 (0.44)	0.176
Headache (%)	5 (4.03)	0 (0)	2 (5)	7 (3.08)	0.146
Allergic reactions (%)	1 (0.81)	0 (0)	1 (2.5)	2 (0.88)	0.398
Fever (%)	5 (4.03)	1 (1.59)	2 (5)	8 (3.52)	0.624
Total complications (%)				89 (39.21)	

## Discussion

In this study, 227 patients presenting IBS were enrolled. Previously, FMT was reported to reduce IBS symptoms in small-scale samples and short-term follow-up (17–19). Whether FMT can produce long-term effects on IBS has not yet been proven. Herein, the effect of FMT on IBS was studied through a long-term (5-year) follow-up and a large sample size (227 cases). The study endpoints included effective follow-up rates, change of IBS-SSS score, IBS-related quality of life and fatigue, effect on stool frequency, Bristol Stool Scale for IBS-C and IBS-D, and side effects of FMT.

Current evidence suggests that the microbiota of the GI tract could be a significant factor in the etiology of IBS (6). The gut microbiota of IBS patients differs from that of healthy subjects, with the former having low bacterial diversity (dysbiosis), for example (7, 8). Changes in the intestinal environment were hypothesized to induce a compositional imbalance of the gut microbiota, termed “dysbiosis,” which was associated with IBS (9). Consequently, probiotics and antibiotics were studied as potential treatment for IBS (10, 11); however, the scale of improvement in symptoms was limited. FMT provides a creative approach to restore the abnormal gut microbiome in patients with IBS. Our team has treated 2,010 cases of various GI dysfunction diseases including IBS through FMT, and the resulting long-term (36 months) efficacy rates were >60% (16). Although the current clinical studies have confirmed the efficacy of FMT in the treatment of IBS, these were short-term studies with small sample sizes; therefore, large-scale long-term studies are still lacking in this field (26).

In 2017, the first randomized controlled trial (RCT) on FMT treatment for IBS was conducted in Norway. Patients were assigned to a group (*n* = 60) comprising subjects who received 50–80 g of fresh FMT (used on the same day) or frozen FMT and a group (*n* = 30) consisting of subjects who received his or her own feces as placebo. Transplantation was performed with colonoscopy. After 3 months of FMT treatment, the IBS-SSS scores decreased by more than 75 points for 36 out of 55 subjects who were actively treated (65%) and 12 out of 28 subjects who received placebo (43%) (*p* = 0.049), indicating that the therapeutic efficacy was significantly better in the treatment group than in the placebo group (27). Since then, in several other randomized controlled studies that have been established, FMT has appeared to be effective at improving the symptoms (IBS-SSS) and the quality of life of patients with IBS, as well as reducing their fatigue (25, 28).

At present, FMT can be administered through a variety of methods, such as oral fecal capsules, nasointestinal injection, or endoscopy. Due to the bacterial overgrowth in the small intestine of IBS patients (29), the upper GI route is more recommended. In this study, we compared the following three methods to treat IBS: nasointestinal tube, capsule, and colonoscopy. The results showed that the capsule approach had the most obvious advantages. The main reason might be that the implementation of this approach is simpler and more convenient and has better medical compliance. Nonetheless, no significant differences were observed concerning the adverse events among the three groups.

Microbiota transplantation has been reported to have significant effects within the 1st day after administration (30), while the engraftment of transferred microbiota may take at least 7 months after FMT (31). The decline of donor strain populations has been detected within 1.5–3 months after FMT (32), and 39 ± 23% of the species showed resistance to introduced strains. Along with the decline of donor strains, the theoretical effect of FMT will also decrease significantly (32). The study by Johnsen et al. showed that, after 3 months of treatment, the efficacy rate of FMT was 65% (36 cases), while treatment response was observed in 12 cases (43%) of the placebo group. There was a significant statistical difference between the FMT and the placebo groups (*p* = 0.049). However, after 12 months of FMT, its effect decreased, and it had a similar effect on participants as the placebo (FMT vs. placebo groups, *p* = 0.075) (28). Therefore, repeated FMT treatments might be required. Previous research showed that a high-dose transplant and/or repeated FMT for IBS may increase the response rate and the intensity of the effects of FMT (25). In our previous clinical studies, we had observed that, following the FMT treatment period, the response decreased over time. Therefore, a repetitive and periodic FMT treatment strategy was subsequently established (33). Herein, it was confirmed that repeated and periodic FMT treatment can significantly ensure the long-term efficacy of FMT.

The present study indicated that the average course of FMT was 3.93 ± 2.30, including 3.83 ± 1.78 for nasointestinal tube patients, 5.19 ± 2.94 for capsule patients, and 2.25 ± 1.24 for colonoscopy patients. The reason behind the larger number of capsule transplants is that it is a simple, non-invasive, and easy route to implement, which leads to better medical compliance. Due to the trauma and discomfort of the nasointestinal tube and colonoscopy, their medical compliance is poor, and the frequency of repeated treatments is limited. Consequently, the good patient compliance and high repetition rate of capsule transplantation may be the main reasons for its high efficacy.

Adverse reactions to FMT treatment should also be addressed. It has been shown that most of these events are GI symptoms, as most patients experience transient diarrhea after FMT treatment, and a few may manifest symptoms such as bloating and belching that usually disappear after 2–3 days (34). In this study, 89 (39.21%) adverse reactions occurred during follow-up. The most common of these were abdominal pain, abdominal distension/bloating, diarrhea, nausea, vomiting, headache, allergic reactions, and fever. The capsules had the least side effects when compared to the nasointestinal tube and colonoscopy. All side effects were reduced by symptomatic treatment, and no serious adverse events occurred during the follow-up period. In 2019, the U.S. Food and Drug Administration (FDA) issued a warning that two donors who had not been tested for multidrug-resistant bacteria caused severe infections after FMT, and one patient died as a result (35). Our team has administered FMT therapy in 5,757 cases of various diseases, and no deaths have occurred. Donors have been tested for multidrug-resistant bacteria and resistant genes since the beginning of the study. At the same time, recipients are being selected according to rigorous standards. In addition to routine tests, we also evaluate the immune function of the recipient, such as lymphocyte count and T lymphocyte subgroup, since patients with immunodeficiency are extremely prone to enteric infections.

Certain limitations of this study need to be highlighted. First, it is a retrospective analysis rather than a prospective randomized controlled study. Second, we mainly focused on the clinical symptoms of IBS patients after FMT but did not follow up the changes of intestinal flora after the treatment period. Extensive research has shown that FMT improves symptoms in patients with IBS by improving the intestinal flora. However, one study indicated that the intestinal flora of FMT significantly enhanced, while the symptoms of IBS did not show any improvement (17).

## Conclusion

In this retrospective study, the effects of three approaches of FMT therapy to treat IBS were evaluated during 5 years of long-term follow-up. The results demonstrated the safety and efficacy of FMT for IBS patients; however, as the treatment effect declines over time, periodic and repetitive treatment is necessary.

## Data Availability Statement

The raw data supporting the conclusions of this article will be made available by the authors, without undue reservation.

## Ethics Statement

The studies involving human participants were reviewed and approved by Ethical Committee of Shanghai Tenth Hospital affiliated to Tongji University. The patients/participants provided their written informed consent to participate in this study.

## Author Contributions

QC, NL, and HQ conceived and designed the study. JC and CY conducted the data collections. Analysis and interpretation of data were done by ZL, HT, BY, and DZ. Statistical analysis was done by QC and JC. Writing and revision of the manuscript were done by JC, ZL, and QC. All authors read and approved the final manuscript.

## Conflict of Interest

The authors declare that the research was conducted in the absence of any commercial or financial relationships that could be construed as a potential conflict of interest.

## Publisher's Note

All claims expressed in this article are solely those of the authors and do not necessarily represent those of their affiliated organizations, or those of the publisher, the editors and the reviewers. Any product that may be evaluated in this article, or claim that may be made by its manufacturer, is not guaranteed or endorsed by the publisher.

## References

[1] Chey WD Kurlander J Eswaran S. Irritable bowel syndrome: a clinical review. JAMA. (2015) 313:949–58. 10.1001/jama.2015.0954 25734736

[2] Lovell RM Ford AC. Global prevalence of and risk factors for irritable bowel syndrome: a meta-analysis. Clin Gastroenterol Hepatol. (2012) 10:712–21. 10.1016/j.cgh.2012.02.029 22426087

[3] Soares RL. Irritable bowel syndrome: a clinical review. World J Gastroenterol. (2014) 20:12144–60. 10.3748/wjg.v20.i34.12144 25232249 PMC4161800

[4] Longstreth GF Thompson WG Chey WD Houghton LA Mearin F Spiller RC. Functional bowel disorders. Gastroenterology. (2006) 130:1480–91. 10.1053/j.gastro.2005.11.061 16678561

[5] Zhang F Xiang W Li CY Li SC. Economic burden of irritable bowel syndrome in China. World J Gastroenterol. (2016) 22:10450–60. 10.3748/wjg.v22.i47.10450 28058026 PMC5175258

[6] Lee KN Lee OY. Intestinal microbiota in pathophysiology and management of irritable bowel syndrome. World J Gastroenterol. (2014) 20:8886–97. 10.3748/wjg.v20.i10.2456 25083061 PMC4112865

[7] Casen C Vebo HC Sekelja M Hegge FT Karlsson MK Ciemniejewska E. Deviations in human gut microbiota: a novel diagnostic test for determining dysbiosis in patients with IBS or IBD. Aliment Pharmacol Ther. (2015) 42:71–83. 10.1111/apt.13236 25973666 PMC5029765

[8] El-Salhy M Mazzawi T. Fecal microbiota transplantation for managing irritable bowel syndrome. Expert Rev Gastroenterol Hepatol. (2018) 12:439–45. 10.1080/17474124.2018.1447380 29493330

[9] Barbara G Feinle-Bisset C Ghoshal UC Quigley EM Santos J Vanner S. The intestinal microenvironment and functional gastrointestinal disorders. Gastroenterology. (2016) 150:1305–1318. 10.1053/j.gastro.2016.02.028 27144620

[10] Lee KJ Tack J. Altered intestinal microbiota in irritable bowel syndrome. Neurogastroenterol Motil. (2010) 22:493–8. 10.1111/j.1365-2982.2010.01482.x 20414959

[11] Pimentel M Lembo A Chey WD Zakko S Ringel Y Yu J. Rifaximin therapy for patients with irritable bowel syndrome without constipation. N Engl J Med. (2011) 364:22–32. 10.1056/NEJMoa1004409 21208106

[12] Mugie SM Di Lorenzo C Benninga MA. Constipation in childhood. Nat Rev Gastroenterol Hepatol. (2011) 8:502–11. 10.1038/nrgastro.2011.130 21808283

[13] van Nood E Vrieze A Nieuwdorp M Fuentes S Zoetendal EG de Vos WM. Duodenal infusion of donor feces for recurrent Clostridium difficile. N Engl J Med. (2013) 368:407–15. 10.1056/NEJMoa1205037 23323867

[14] Lee CH Steiner T Petrof EO Smieja M Roscoe D Nematallah A. Frozen vs fresh fecal microbiota transplantation and clinical resolution of diarrhea in patients with recurrent clostridium difficile infection: a randomized clinical trial. JAMA. (2016) 315:142–9. 10.1001/jama.2015.18098 26757463

[15] Khoruts A Dicksved J Jansson JK Sadowsky MJ. Changes in the composition of the human fecal microbiome after bacteriotherapy for recurrent Clostridium difficile-associated diarrhea. J Clin Gastroenterol. (2010) 44:354–60. 10.1097/MCG.0b013e3181c87e02 20048681

[16] Li N Tian HL Chen QY Yang B Ma CL Lin ZL. [Efficacy analysis of fecal microbiota transplantation in the treatment of 2010 patients with intestinal disorders]. Zhonghua Wei Chang Wai Ke Za Zhi. (2019) 22:861–8. 10.3760/cma.j.issn.1671-0274.2019.09.011 31550826

[17] Halkjaer SI Christensen AH Lo BZS Browne PD Gunther S Hansen LH. Faecal microbiota transplantation alters gut microbiota in patients with irritable bowel syndrome: results from a randomised, double-blind placebo-controlled study. Gut. (2018) 67:2107–15. 10.1136/gutjnl-2018-316434 29980607

[18] Aroniadis OC Brandt LJ Oneto C Feuerstadt P Sherman A Wolkoff AW. Faecal microbiota transplantation for diarrhoea-predominant irritable bowel syndrome: a double-blind, randomised, placebo-controlled trial. Lancet Gastroenterol Hepatol. (2019) 4:675–85. 10.1016/S2468-1253(19)30198-0 31326345

[19] El-Salhy M Hatlebakk JG Gilja OH Brathen Kristoffersen A Hausken T. Efficacy of faecal microbiota transplantation for patients with irritable bowel syndrome in a randomised, double-blind, placebo-controlled study. Gut. (2020) 69:859–67. 10.1136/gutjnl-2019-319630 31852769 PMC7229896

[20] Trotti A Colevas AD Setser A Rusch V Jaques D Budach V. CTCAE v3.0: development of a comprehensive grading system for the adverse effects of cancer treatment. Semin Radiat Oncol. (2003) 13:176–81. 10.1016/S1053-4296(03)00031-6 12903007

[21] Cammarota G Ianiro G Tilg H Rajilic-Stojanovic M Kump P Satokari R. European consensus conference on faecal microbiota transplantation in clinical practice. Gut. (2017) 66:569–80. 10.1136/gutjnl-2016-313017 28087657 PMC5529972

[22] Mullish BH Quraishi MN Segal JP McCune VL Baxter M Marsden GL. The use of faecal microbiota transplant as treatment for recurrent or refractory Clostridium difficile infection and other potential indications: joint British Society of Gastroenterology (BSG) and Healthcare Infection Society (HIS) guidelines. Gut. (2018) 67:1920–41. 10.1136/gutjnl-2018-316818 30154172

[23] Tian H Ge X Nie Y Yang L Ding C McFarland LV. Fecal microbiota transplantation in patients with slow-transit constipation: a randomized, clinical trial. PLoS ONE. (2017) 12:e0171308. 10.1371/journal.pone.0171308 28158276 PMC5291446

[24] Tian H Ding C Gong J Wei Y McFarland LV Li N. Freeze-dried, Capsulized Fecal Microbiota Transplantation for Relapsing Clostridium difficile Infection. J Clin Gastroenterol. (2015) 49:537–8. 10.1097/MCG.0000000000000330 25955501

[25] El-Salhy M Hausken T Hatlebakk JG. Increasing the dose and/or repeating faecal microbiota transplantation (FMT) increases the response in patients with irritable bowel syndrome (IBS). Nutrients. (2019) 11:1415. 10.3390/nu11061415 31238507 PMC6628324

[26] Wang Y Zheng F Liu S Luo H. Research progress in fecal microbiota transplantation as treatment for irritable bowel syndrome. Gastroenterol Res Pract. (2019) 2019:9759138. 10.1155/2019/9759138 31885549 PMC6914991

[27] Ford AC. Stool as a treatment for IBS: more questions than answers? Lancet Gastroenterol Hepatol. (2018) 3:2–3. 10.1016/S2468-1253(17)30337-0 29100844

[28] Johnsen PH Hilpusch F Cavanagh JP Leikanger IS Kolstad C Valle PC. Faecal microbiota transplantation versus placebo for moderate-to-severe irritable bowel syndrome: a double-blind, randomised, placebo-controlled, parallel-group, single-centre trial. Lancet Gastroenterol Hepatol. (2018) 3:17–24. 10.1016/S2468-1253(17)30338-2 29100842

[29] Wu KQ Sun WJ Li N Chen YQ Wei YL Chen DF. Small intestinal bacterial overgrowth is associated with Diarrhea-predominant irritable bowel syndrome by increasing mainly Prevotella abundance. Scand J Gastroenterol. (2019) 54:1419–25. 10.1080/00365521.2019.1694067 31765575

[30] Jalanka J Mattila E Jouhten H Hartman J de Vos WM Arkkila P. Long-term effects on luminal and mucosal microbiota and commonly acquired taxa in faecal microbiota transplantation for recurrent Clostridium difficile infection. BMC Med. (2016) 14:155. 10.1186/s12916-016-0698-z 27724956 PMC5057499

[31] Broecker F Klumpp J Schuppler M Russo G Biedermann L Hombach M. Long-term changes of bacterial and viral compositions in the intestine of a recovered Clostridium difficile patient after fecal microbiota transplantation. Cold Spring Harb Mol Case Stud. (2016) 2:a000448. 10.1101/mcs.a000448 27148577 PMC4849847

[32] Li SS Zhu A Benes V Costea PI Hercog R Hildebrand F. Durable coexistence of donor and recipient strains after fecal microbiota transplantation. Science. (2016) 352:586–9. 10.1126/science.aad8852 27126044

[33] Zhang X Tian H Ma C Yang B Hua Y Zhu Y. [Efficacy observation of periodic fecal microbiota transplantation in the treatment of refractory constipation]. Zhonghua Wei Chang Wai Ke Za Zhi. (2017) 20:1355–9. 10.3760/cma.j.issn.1671-0274.2017.12.008 29280116

[34] Jiang ZD Ajami NJ Petrosino JF Jun G Hanis CL Shah M. Randomised clinical trial: faecal microbiota transplantation for recurrent Clostridum difficile infection - fresh, or frozen, or lyophilised microbiota from a small pool of healthy donors delivered by colonoscopy. Aliment Pharmacol Ther. (2017) 45:899–908. 10.1111/apt.13969 28220514

[35] DeFilipp Z Bloom PP Torres Soto M Mansour MK Sater MRA Huntley MH. Drug-resistant *E. coli* bacteremia transmitted by fecal microbiota transplant. N Engl J Med. (2019) 381:2043–50. 10.1056/NEJMoa1910437 31665575

